# Mining Bovine Milk Proteins for DPP-4 Inhibitory Peptides Using Machine Learning and Virtual Proteolysis

**DOI:** 10.34133/research.0391

**Published:** 2024-06-17

**Authors:** Yiyun Zhang, Yiqing Zhu, Xin Bao, Zijian Dai, Qun Shen, Liyang Wang, Yong Xue

**Affiliations:** ^1^ National Engineering and Technology Research Center for Fruits and Vegetables, College of Food Science and Nutritional Engineering, China Agricultural University, Beijing 100083, P.R. China.; ^2^ National Center of Technology Innovation (Deep Processing of Highland Barley) in Food Industry, China Agricultural University, Haidian District, Beijing 100083, P.R. China.; ^3^ School of Clinical Medicine, Tsinghua University, Beijing 100084, P.R. China.

## Abstract

Dipeptidyl peptidase-IV (DPP-4) enzyme inhibitors are a promising category of diabetes medications. Bioactive peptides, particularly those derived from bovine milk proteins, play crucial roles in inhibiting the DPP-4 enzyme. This study describes a comprehensive strategy for DPP-4 inhibitory peptide discovery and validation that combines machine learning and virtual proteolysis techniques. Five machine learning models, including GBDT, XGBoost, LightGBM, CatBoost, and RF, were trained. Notably, LightGBM demonstrated superior performance with an AUC value of 0.92 ± 0.01. Subsequently, LightGBM was employed to forecast the DPP-4 inhibitory potential of peptides generated through virtual proteolysis of milk proteins. Through a series of in silico screening process and in vitro experiments, GPVRGPF and HPHPHL were found to exhibit good DPP-4 inhibitory activity. Molecular docking and molecular dynamics simulations further confirmed the inhibitory mechanisms of these peptides. Through retracing the virtual proteolysis steps, it was found that GPVRGPF can be obtained from β-casein through enzymatic hydrolysis by chymotrypsin, while HPHPHL can be obtained from κ-casein through enzymatic hydrolysis by stem bromelain or papain. In summary, the integration of machine learning and virtual proteolysis techniques can aid in the preliminary determination of key hydrolysis parameters and facilitate the efficient screening of bioactive peptides.

## Introduction

Diabetes greatly contributes to global mortality and disability; its prevalence has increased over the past few decades, constituting a substantial worldwide public health challenge [1]. Dietary interventions have emerged as pivotal components in diabetes management, and bioactive peptides derived from food have important roles in health and disease [2]. Evidence from epidemiological studies has consistently revealed a discernible inverse relationship between dairy consumption and diabetes prevalence [3,4], which is likely related to bioactive peptides derived from milk [5]. Among them, dipeptidyl peptidase IV (DPP-4) inhibitory peptides may be key substances through which milk exerts its hypoglycemic effect [6,7]. DPP-4 is a metabolic enzyme that cleaves and deactivates glucagon-like peptide-1 and glucose inhibitory polypeptides during the postprandial phase, reducing their insulinotropic effects [8]. Therefore, DPP-4 enzyme inhibitors are a promising category of diabetes medications [9].

There are significant challenges associated with the preparation of bioactive peptides. These challenges stem from the diversity of the original proteins and the complexity of the structural peptides in the hydrolysis mixture. As a result, producing bioactive peptides that are high in content, structurally clear, and functionally distinct becomes difficult [10]. Common methods involve the direct synthesis of specific bioactive peptides. However, this approach is expensive, labor-intensive, and time-consuming [11], rendering it less practical for functional food production. Hence, it is essential to shift the research focus from complex and uncontrollable proteins to proteins with clear structures and well-defined sequences. Within the realm of industrial-scale peptide production, the technology for isolating milk proteins, including milk protein isolates, is well-established. Advanced separation techniques, such as ultrafiltration and microfiltration, can precisely separate casein and whey proteins [12], each of which can be further separated into individual proteins such as α-S1-casein, α-S2-casein, β-casein, and κ-casein, among others [5], all of whose sequences are known. Technological advancements have aided the collection of a substantial amount of milk protein data that are available in public databases, including UniProt, the Milk Protein Database, and the Protein Data Bank (PDB), making it more convenient for researchers to access and utilize relevant information.

Determination of hydrolysis parameters is a crucial step in the study and development of bioactive peptides with desired characteristics. The commonly used methods for determining hydrolysis parameters are response surface methodology and one-factor-at-a-time optimization, which typically involve high experimental and resource costs, are time-consuming, and may not effectively explore the entire parameter space [13]. More recently, virtual proteolysis has emerged as a popular choice in the peptide field. Through virtual proteolysis, it is possible to simulate the hydrolysis process of different proteins under the influence of various enzymes [14]. This aids in swiftly obtaining information about the proteins and enzymes capable of generating specific peptides. Virtual proteolysis technology has gradually been applied in the food industry. For example, virtual proteolysis was used to identify peptides in egg whites that could serve as xanthine oxidase inhibitors [15], and antihypertensive oligopeptides were identified in adlay through virtual proteolysis and virtual screening [16]. Conventional approaches for peptide screening typically include in vitro assays that are both time-consuming and expensive and do not consistently ensure the candidate peptide discovery. The use of virtual screening techniques, such as molecular docking, involved several limitations, including limited sampling of ligand and receptor conformations in pose prediction as well as imprecise scoring algorithms that may result in outcomes that do not align with experimental binding affinities [17]. Given the need for the efficient and high-throughput screening of bioactive peptides derived from food, machine learning has emerged as a promising solution. Machine learning involves the selection of optimal algorithms through extensive analysis of historical data, enabling it to make accurate predictions [18]. Some studies have successfully employed machine learning to discover new DPP-4 inhibitory peptides, although these discoveries have not originated from food sources. For example, machine learning techniques, including naive Bayesian and recursive partitioning, were used to predict DPP-4 inhibitors through the construction of 247 models using a dataset of 1,307 known DPP-4 inhibitors and achieved predictive accuracies above 80% [19]. Additionally, an easily interpretable sequence-based predictor called iDPPIV-SCM was developed to predict the DPP-4 inhibitory bioactivity of peptides [20]. Therefore, efficient screening of potential bioactive peptides and preliminary determination of key hydrolysis parameters based on virtual proteolysis and machine learning technologies is a feasible pathway toward achieving high-quality preparation of bioactive peptides from food sources.

**Fig. 1.**
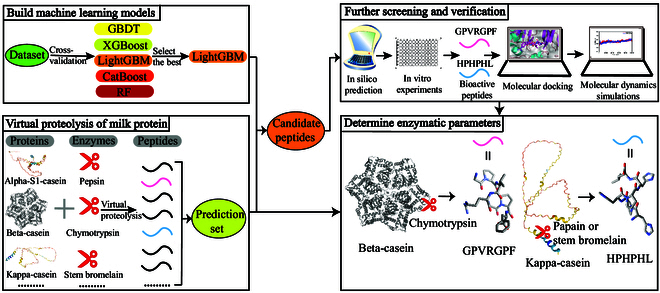
Schematic showing the experimental workflow for identifying DPP-4 inhibitory peptides from milk proteins. This framework includes four steps: (1) Building machine learning models and selecting the best-performing model for subsequent predictions. (2) Virtual proteolysis of different milk proteins and enzymes helps to obtain numerous peptides used as input for the machine learning model. Peptides predicted by the model to have DPP-4 inhibitory activity are then chosen as candidate peptides. (3) Further selection of target peptides from candidate peptides through in silico prediction and in vitro experiments, followed by validation through molecular docking and molecular dynamics simulations. (4) By retrospectively examining the virtual proteolysis part, main hydrolysis parameters including the selection of protein and enzyme are preliminarily determined. The five machine learning models included Gradient Boosted Decision Trees (GBDT), Extreme Gradient Boosting (XGBoost), Light Gradient Boosting Machine (LightGBM), Categorical Boosting (CatBoost), and Random Forest (RF).

Therefore, in this study, bovine milk protein was used as a raw material to develop a novel screening framework that combines virtual proteolysis and machine learning technologies to identify DPP-4 inhibitory peptides and preliminarily determine main hydrolysis parameters, as shown in Fig. 1. The findings were further validated by a series of in vitro experiments, molecular docking, and molecular dynamics simulations. This innovative approach not only saves cost and time in the discovery of bioactive peptides but also promotes the precise preparation of bioactive peptides.

## Results

### Result of the dataset

**Fig. 2.**
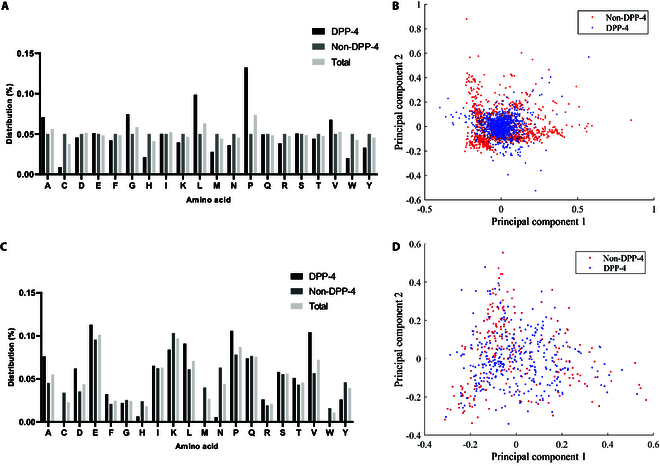
Results of the training and prediction sets. (A) Frequency distribution of amino acids in the training set. (B) Principal component scatterplot of the training set. (C) Frequency distribution of amino acids in the prediction set. (D) Principal component scatterplot of the prediction set. A, alanine (Ala); R, arginine (Arg); N, asparagine (Asn); D, aspartic acid (Asp); C, cysteine (Cys); Q, glutamine (Gln); E, glutamic acid (Glu); G, glycine (Gly); I, isoleucine (Ile); L, leucine (Leu); K, lysine (Lys); M, methionine (Met); F, phenylalanine (Phe); P, proline (Pro); S, serine (Ser); T, threonine (Thr); W, tryptophan (Trp); Y, tyrosine (Tyr); V, valine (Val).

Amino acid composition can be intricately linked to the biological activity of peptides [21]. Therefore, the amino acid distribution of DPP-4 inhibitory and non-DPP-4 inhibitory peptides in the training and prediction sets was analyzed. In the training set, certain hydrophobic amino acids including alanine (Ala), glycine (Gly), leucine (Leu), proline (Pro), and valine (Val) were enriched in DPP-4 inhibitory peptides, compared with non-DPP-4 inhibitory peptides, which were randomly extracted and had uniform amino acid distribution. In the prediction set derived from the virtual hydrolysis of milk proteins, Ala, glutamate (Glu), Pro, and Val were abundant in the peptides predicted to inhibit DPP-4. In this work, the principal components analysis (PCA) method is applied to the training and prediction sets to observe the distinctions of positive and negative data distributions. First, the training and prediction sets are decentralized, and then, two transformation matrixes corresponding to the training and prediction sets, respectively, are determined to find the projection of the sets, in order to accomplish the dimensionality reduction. To observe the distributions in two dimensionality space, two principal components projected by PCA on all the features of each set are preserved and treated to be the two-axis shown as Fig. 2B and D. It is shown that a distinction was noted in the distribution of DPP-4 inhibitory peptides and non-DPP-4 inhibitory peptides within the training and prediction sets.

### Performance of the machine learning models

**Fig. 3.**
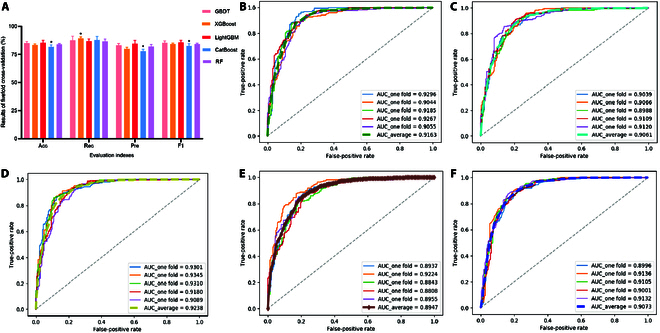
Results of machine learning models. (A) Evaluation indexes of the machine learning models. The results show the average value of each index, where Acc, Rec, Pre, and F1 represent accuracy, recall, precision, and F1 score, respectively. The error bars represent means ± SD. The asterisk (*) indicates a significant difference compared to the LightGBM model at a significance level of *P* < 0.05. (B) ROC curves and AUC values of GBDT. ROC curves and AUC values of (C) XGBoost, (D) LightGBM, (E) CatBoost, and (F) RF.

To determine the most suitable model for predicting DPP-4 inhibitory peptides, five different ensemble models were trained and validated (Fig. 3 and Table S1). Using fivefold cross-validation, we found that the area under the curve (AUC) of Light Gradient Boosting Machine (LightGBM) was higher than that of the other four models, indicating its superior classification ability. Furthermore, the accuracy (Acc) of the LightGBM model outperformed the other machine learning models and was significantly higher than that of the Categorical Boosting (CatBoost) model (*P* < 0.05), indicating its stronger generalization ability. The precision (Pre) and F1 scores (F1) of the LightGBM model were 84.62 and 85.70, respectively, both of which were higher than those of the other four models and significantly higher than those of the CatBoost model (*P* < 0.05), further emphasizing its higher stability. However, the recall (Rec) value from the LightGBM model was less than that of the Extreme Gradient Boosting (XGBoost) model (*P* < 0.05). Overall, compared with the other four models, LightGBM showed better overall performance and was selected for DPP-4 inhibitory peptide screening.

### In silico prediction and IC_50_ values for selected peptide candidates

By conducting virtual hydrolysis of bovine milk proteins, a total of 737 peptides were obtained. Initially, the LightGBM model was employed to identify potential DPP-4 inhibitory peptides. Subsequently, for the future development and application of peptides, in silico predictions of potential DPP-4 inhibitory peptides were conducted, including the comprehensive biological activity prediction, human intestinal absorption (HIA) prediction, and toxicity prediction using PeptidRanker, AdmetSAR, and ToxinPred tools, respectively. Peptides with high biological activity, good intestinal absorption, and nontoxicity were selected, resulting in 37 peptides. Then, three peptides were randomly selected for in vitro experiments. The in silico prediction results and IC_50_ values of these three peptides were presented in Table. The inhibitory effects of GPVRGPF and HPHPHL on DPP-4 enzyme are significant, with IC_50_ values of 0.47 mg/ml (645 μM) and 1.19 mg/ml (1,614 μM), respectively. However, FVAPFPEV exhibits poor inhibitory effect. Compared to previous studies, for example, the IC_50_ values of GL and AL found in milk proteins were 2,615.03 ± 612.80 μM and 882.13 ± 68.66 μM, respectively [22], and the IC_50_ values of TPEVDDEALEK and VPL were 320 and 16 μM, respectively [6], whereas the IC_50_ values of GPVRGPF and HPHPHL found in this work were 645 and 1,614 μM, respectively, which are at an intermediate level. It is important to note that direct comparisons between these peptides can be challenging because of differences in experimental parameters and methodologies among studies. Besides, by retracing the virtual proteolysis conditions, we found that GPVRGPF was obtained from β-casein through enzymatic hydrolysis by chymotrypsin, and HPHPHL was obtained from κ-casein through enzymatic hydrolysis by stem bromelain or papain.

**Table.** In silico prediction and IC_50_ value determination for peptides

Sequence	Protein	Enzyme	PeptideRanker score	Toxicity	Human intestinal absorption	IC_50_ (μM)
GPVRGPF	β-Casein	Chymotrypsin	0.878	Nontoxic	HIA+	645
HPHPHL	κ-Casein	Stem bromelain, papain	0.748	Nontoxic	HIA+	1,614
FVAPFPEV	α-S1-casein	Pepsin (pH 1.3), pepsin (pH > 2)	0.518	Nontoxic	HIA+	\

The PeptideRanker score indicates the bioactivity level of a peptide as determined by the PeptideRanker tool. Peptides with scores closer to 1.0 are predicted to have higher bioactive potential. Toxicity refers to the prediction results generated by the ToxinPred tool, which indicate whether a peptide is likely to be toxic or nontoxic. HIA+ indicates that the peptide has good intestinal absorption, which is predicted by the AdmetSAR tool.

### Molecular docking of candidate peptides and DPP-4

Next, we used molecular docking to clarify the binding mode, binding sites, and binding affinities between DPP-4 and the GPVRGPF and HPHPHL peptides, which demonstrated DPP-4 inhibitory effects in vitro. Table S2 shows the docking energy scoring and the interactions between the peptides and DPP-4, including the participating residues. According to the predictions from the AutoDock4 software, the binding energies of the GPVRGPF and HPHPHL peptides with DPP-4 were −9.56 and −10.05 kcal/mol, respectively. The negative binding energies indicated that, theoretically, these two peptides can spontaneously bind to DPP-4.

**Fig. 4.**
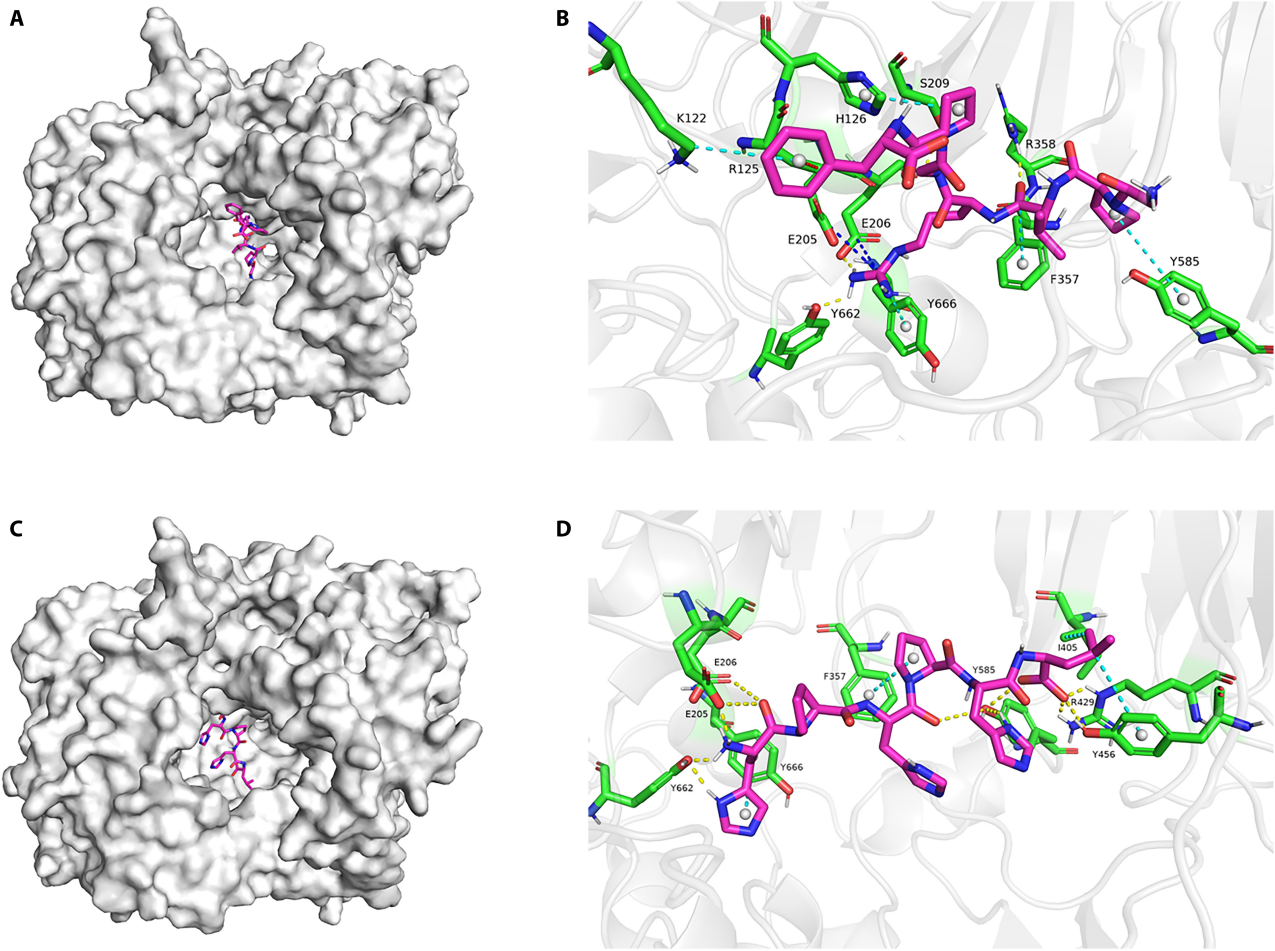
Results of molecule docking. The (A) overall and (B) local schematics of the GPVRGPF–DPP-4 system. The (C) overall and (D) local schematics of the HPHPHL–DPP-4 system. In these diagrams, yellow dashed lines represent hydrogen bonds, blue dashed lines represent salt bridges, indigo dashed lines represent hydrophobic interactions, and red dashed lines represent π-stacking. The protein is depicted using as a cartoon model, while the binding site residues and peptides are represented using stick models.

DPP-4 contains a cavity-shaped active site that inhibitors typically compete to occupy by binding to residues (S1, S2, and S3) located within the cavity. The molecular docking results showed that both peptides competitively occupied the active site by binding to residues S1, S2, and S3 (Fig. 4). According to the PLIP and LigPlot+ analyses, the HPHPHL peptide sequence interacted with the DPP-4 active site by forming hydrogen bonds, salt bridges, π-stacking, and hydrophobic interactions with Ser^630^, Tyr^631^, Tyr^662^, Tyr^666^, and Asn^710^ in S1, Tyr^662^ in S2, and Phe^357^ in S3. Similarly, the GPVRGPF peptide sequence formed hydrogen bonds, salt bridges, π-stacking, and hydrophobic interactions with Tyr^662^ and Tyr^666^ in S1, Glu^205^, Glu^206^, and Tyr^662^ in S2, and Ser^209^, Arg^358^, and Phe^357^ in S3 within the active site of DPP-4. Overall, the docking predictions suggested that both GPVRGPF and HPHPHL peptide sequences could inhibit the activity of DPP-4 by interacting with its active pocket.

### Molecular dynamics simulation of DPP-4–peptide complexes

**Fig. 5.**
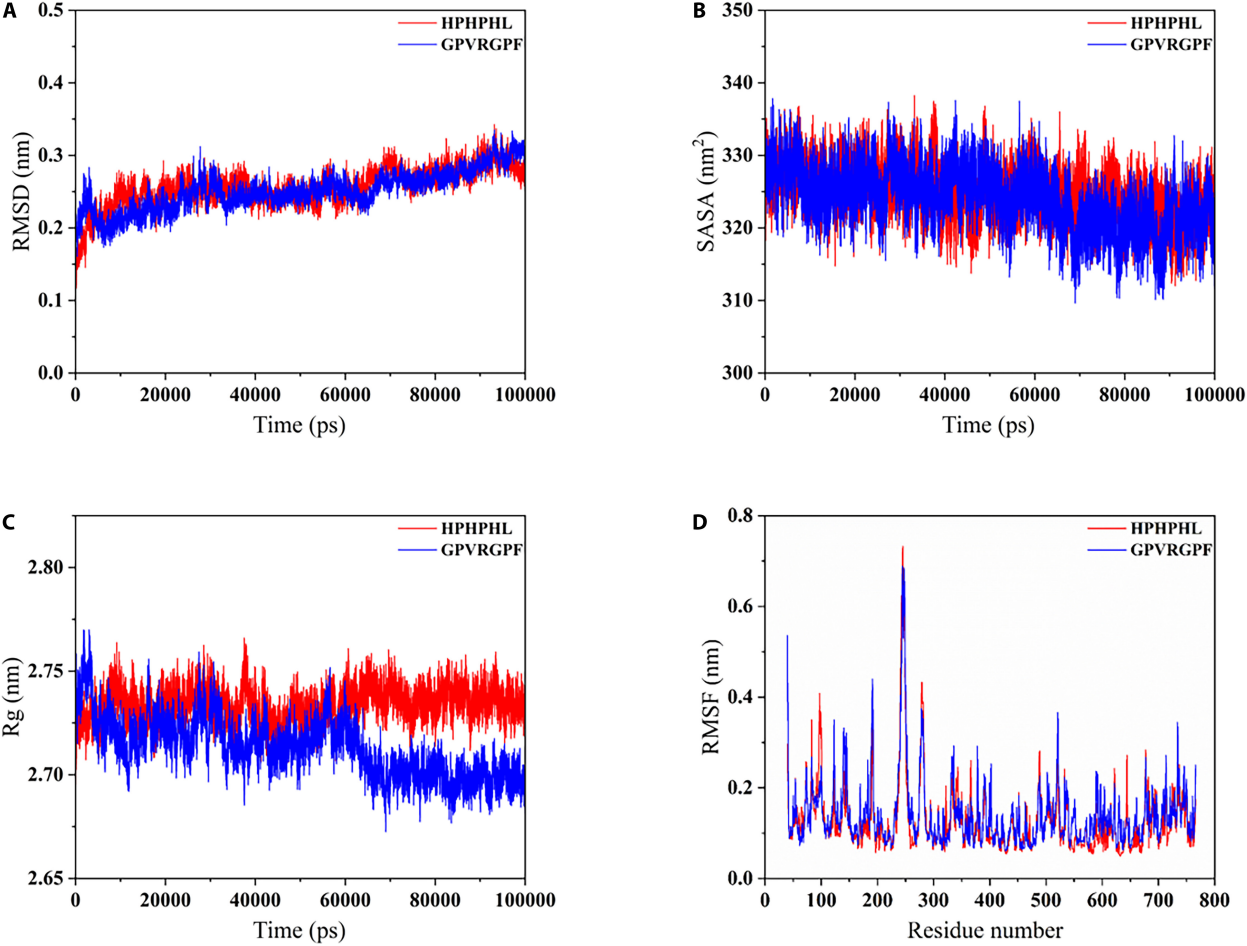
Molecular dynamics simulation of GPVRGPF–DPP-4 and HPHPHL–DPP-4 complexes. (A) Root mean square deviation (RMSD) of the main chain of DPP-4 with the peptides. (B) Solvent-accessible surface area (SASA) values. (C) Radius of gyration (Rg) and (D) root mean square fluctuation (RMSF) of DPP-4, respectively. Red, HPHPHL; blue, GPVRGPF.

To further elucidate the stability and flexibility of the DPP-4–peptide complexes, as well as the impact of dynamic motion on their binding interactions, molecular dynamics simulations were performed with both GPVRGPF and HPHPHL. After DPP-4 bound with both peptides, the root mean square deviation (RMSD) values of its main-chain structure ranged from 0.2 to 0.3 nm, with fluctuations within 100 ns that did not exceed 0.1 nm, indicating that the DPP-4–peptide complex structures were stable (Fig. 5). Furthermore, the GPVRGPF–DPP-4 system exhibited smaller fluctuations in the DPP-4 main-chain structure, suggesting higher stability in its protein structural conformation.

Upon binding of the peptides to the DPP-4 active pocket region, the solvent-accessible surface area (SASA) values of DPP-4 decreased, indicating that the binding of both peptides promoted tight structural changes in DPP-4. The radius of gyration (Rg) value of DPP-4 in the HPHPHL system remained largely unchanged, while that of the GPVRGPF system decreased. This suggested that HPHPHL had a relatively small impact on the DPP-4 structure, while GPVRGPF had a more significant effect. Overall, the results indicate that GPVRGPF binding to DPP-4 was dependent on changes in the DPP-4 structure. Additionally, the root mean square fluctuation (RMSF) values of DPP-4 showed significant residue fluctuations in the GPVRGPF system, consistent with our other findings and further suggesting that the binding of GPVRGPF to DPP-4 depends on induced changes in the DPP-4 structure.

## Discussion

This study developed an efficient framework for screening DPP-4 inhibitory peptides using virtual proteolysis and machine learning technologies. We successfully discovered two novel DPP-4 inhibitory peptides (GPVRGPF and HPHPHL) from milk proteins that exhibited good in vitro DPP-4 inhibitory activities. Molecular docking and dynamics simulations indicated that GPVRGPF and HPHPHL interacted with the DPP-4 active site and induced structural changes in DPP-4. In addition, the conventional method for identifying bioactive peptides involves in vitro digestion and chromatographic purification of the hydrolyzed product, followed by bioactivity assessment and sequence identification, which is often time-consuming and expensive [21]. In addition, further optimization of hydrolysis parameters is needed. Our research framework allows for the simultaneous prediction of multiple samples, resulting in significant cost and time savings. Furthermore, this framework can preliminarily identify key parameters for obtaining specific bioactive peptides including the selection of protein and enzyme, which is of significant importance for the precise preparation of bioactive peptides.

To the prospect of future, it is valuable to investigate the improved feature representation methods to enhance the performance of the classification models. Recently, many methods are devised to improve the feature representation ability of the dataset. For instance, the developed tabular data techniques, such as DeepInsight [23], have received a wide focus, which transform the tabular data into image samples for further model applications. These tabular data techniques are feasible to be the complementary of the conventional approaches for peptide screening: First, the non-image peptide datasets are often characterized by the complicated high-dimensional structures, and the traditional feature extraction approaches are inefficient to capture the underlying patterns. The conversion from non-image samples to image-like representations provides a structured and uniform format that can be processed by further models; second, converting non-image data into images can also enable the existing domain-specific knowledge or pretrained models, and the possibility of leveraging these pretrained models or transfer learning techniques is provided. This method can help us develop higher-performance models in the future.

Machine learning encompasses various branches, such as deep learning and ensemble learning. Ensemble learning is a popular choice in the field of activity prediction because it combines multiple models to enhance predictive performance compared to individual models. This is particularly advantageous for accelerating research on quantitative structure–activity and structure–property relationships [24]. Moreover, compared with deep learning, ensemble learning offers better interpretability, making it easier to understand how predictions are generated. In addition, ensemble learning typically has low computational requirements, making it suitable for resource-constrained environments. However, similar to traditional machine learning, ensemble learning can require manual feature engineering, which is time consuming. Compared with deep learning, ensemble learning can struggle to capture highly complex nonlinear relationships in data [25]. Thus, the choice between ensemble learning, deep learning, and traditional machine learning depends on factors such as the nature of the data, available computational resources, interpretability requirements, and dataset size. In this study, we selected and evaluated several ensemble learning models, including Gradient Boosted Decision Trees (GBDT), XGBoost, LightGBM, CatBoost, and Random Forest (RF), and found that LightGBM exhibited the best performance. Previous studies have also employed machine learning to screen for DPP-4 inhibitory peptides, such as Recursive Partitioning [19], StackDPPIV [26], and iDPPIV-SCM [20]. Comparatively, LightGBM achieved a higher accuracy rate (85.55%) than iDPPIV-SCM (82.00%) and Recursive Partitioning (82.80%), but lower than StackDPPIV (89.10%). StackDPPIV utilizes a stacking ensemble method, which, despite its high accuracy, suffers from large model sizes and redundancy. In contrast, LightGBM undergoes model pruning, ensuring both high accuracy and lightweight model construction, resulting in faster training speeds and reduced memory requirements. Furthermore, LightGBM exhibits exceptional efficiency and scalability, rendering it particularly adept at handling large and complex datasets that are crucial for bioinformatics applications [27]. This efficiency allows LightGBM to capture intricate patterns and relationships within the data, which can be missed by other models. The leafwise growth strategy and gradient-based optimization techniques of LightGBM enable the construction of more informative and deeper trees, enhancing its predictive power [28]. The efficient computational processing and optimized tree structure of LightGBM make it an excellent choice for accurately predicting DPP-4 inhibitory activity of peptides.

In recent years, virtual proteolysis technology has been increasingly applied in the field of food and has made significant progress. Virtual proteolysis can be achieved through computer simulations to rapidly and cost-effectively predict protein hydrolysis products. However, in vitro digestion is closer to the human digestive process, and by simulating the stages of oral, gastric, and intestinal digestion, it can more realistically reflect the changes of proteins during digestion, yielding more reliable results [29]. Therefore, we suggest using our strategy proposed in this study to preliminarily determine the main hydrolysis parameters, and then conduct additional in vitro digestion experiments for validation. Additionally, after proteins are digested, the peptides and amino acids produced will enter cells and participate in various metabolic pathways. Therefore, if further understanding of the metabolism and utilization of amino acids in the human body is needed, or if peptides are to be developed into drugs, research on the amino acid metabolic pathways is also necessary.

Previous studies have demonstrated that enzymatic hydrolysis of whey proteins by proteases can lead to the formation of aggregates [30], which poses a challenge to the practical application of our research strategy. Protein aggregation generally has negative effects on protein hydrolysis, such as reducing hydrolysis efficiency, causing irregular degradation products, and affecting the release of bioactive peptides. In food, protein aggregation can occur in various modes, each of which may have different effects on peptide production and associated hydrolysis, including heat-induced aggregation, pH-induced aggregation, salt-induced aggregation, mechanically induced aggregation, and so on [31]. Therefore, in practical applications, the issue of protein aggregation needs to be considered, and appropriate methods need to be explored to address these issues to ensure effective protein hydrolysis.

This study found that peptides GPVRGPF and HPHPHL can effectively inhibit DPP-4 in vitro. In the future, to promote the use of peptides GPVRGPF and HPHPHL as functional ingredients in the functional food and nutritional supplement industries, or to develop them as supplements for the treatment and management of diabetes, metabolomics research is highly necessary. Metabolomics is a commonly used tool for evaluating the effects of various substances on circulating metabolites in the body, which can help understand the regulation process by bioactive peptides, potential mechanisms of action, and information on regulatory pathways involved in alleviating disease conditions [32]. In addition, comprehensive quality analysis of peptides is needed, including purity, characteristics, structure, stability, solubility, in vivo efficacy, and so on. Peptide purity directly influences biological activity. Higher purity peptides generally exhibit greater biological activity because impurities can interfere with the molecular interactions or functions of the peptide. Besides, solubility and stability of peptides can affect the bioavailability, thereby affecting the efficacy. However, the development of techniques such as encapsulation has gradually overcome these issues. Furthermore, some peptides have been reported to exhibit good effects in vitro but are ineffective in vivo. Therefore, in vivo validation is vital. In summary, in order to facilitate the future applications of peptides, conducting comprehensive evaluation experiments is necessary, representing our future research directions.

This study utilized milk proteins with known sequences as raw materials, and it is also applicable to other food proteins with known sequences, such as egg, soy, and wheat proteins [33]. However, this strategy is not currently applicable to food proteins with incomplete or limited sequences. Application to industrial production must also consider the accessibility and cost of protein. As research in proteomics, genomics, and mass spectrometry continues, more food protein sequences will be identified and contribute to the broader applicability of this research strategy.

## Materials and Methods

### Machine learning model training

#### Dataset construction

In order to get reliable and comprehensive positive samples, a total of 1,103 peptides with known DPP-4 inhibitory activity were collected from the recent literature (published before April 2023) and various public databases, including BIOPEP (bioactive peptide database), MBPDB (milk bioactive peptide database), PlantPepDB (plant peptide database), and DFBP (food-derived bioactive database). Experimentally validated non-DPP-4 inhibitory peptides are lacking, and therefore, a commonly used method was followed [34]. We randomly selected peptides from the UniProt database (https://www.uniprot.org/) and designated them as non-DPP-4 inhibitory activity peptides (negative samples). Although it is possible that some of these peptides may display DPP-4 inhibitory activity, the likelihood of this occurrence was low and did not significantly impact the results. The negative and positive samples constituted the training set used to train the machine learning models (Table S3).

#### Feature representation

In bioinformatics and computational biology, amino acid feature extraction methods are important in protein analysis [11]. One powerful feature extraction method is pseudo amino acid composition (PseAAC) [35]. In contrast to the traditional amino acid composition approach that simply counts the occurrence of different amino acids in a protein sequence, PseAAC is a more sophisticated approach because it considers both the amino acid composition and the order and pattern of amino acids in a sequence. This is accomplished through the incorporation of diverse physicochemical and biochemical properties of amino acids, allowing for richer representation of protein sequences [36]. There are three distinct types of PseAAC, with type 2 chosen for this study. The resulting type 2 PseAAC output consisted of 20 + *n* × *k* discrete values, where *k* is the rank of correlation computed along a protein sequence and *n* is the number of user-selected amino acid properties. We selected properties that encompassed hydrophobicity, hydrophilicity, mass, pK1 (α-CO_2_H), pK2 (NH_3_), and isoelectric point (pI) at 25 °C. The parameter *k* was configured as 1, *n* as 6, and the weight factor ω was set to 0.05. The type 2 PseAAC output was used as the input for machine learning models.

#### Machine learning models

Five ensemble learning models (GBDT, XGBoost, LightGBM, CatBoost, and RF) were used in this study. Peak performance in predicting peptide activity is ensured by fine-tuning the models to a specific dataset.

#### GBDT

GBDT is a popular ensemble learning method for regression and classification, which excels at generating highly accurate predictions by combining multiple decision trees [37]. GBDT has been used to classify the anticancer and non-anticancer peptides [38]. Experiments on independent test sets and 10-fold cross-validation sets demonstrated that the GBDT classifier outperformed the other methods. In this work, the parameter n_estimators was set to 200 with a learning rate of 0.1 to ensure optimal protein–peptide activity predictions.

#### XGBoost

XGBoost is an efficient and scalable implementation of gradient boosting [39]. It is versatile, handles both classification and regression, and is robust against overfitting even with missing data. Currently, Wang et al. [40] used the XGBoost model to predict antihypertensive peptides in milk and validated them using molecular docking. The results showed that the XGBoost model performed excellently with an accuracy of 86.50%. In the present study, we set max_depth to 5, the learning rate to 0.01, and the α value to 10.

#### LightGBM

LightGBM is a powerful gradient-boosting framework for machine learning tasks. It uses a histogram-based approach for tree building, which reduces memory usage and speeds up training [41]. Lv et al. [42] introduced AMPpred-EL as a novel antimicrobial peptide prediction method that utilizes ensemble learning. AMPpred-EL fuses ensemble learning with LightGBM and logistic regression, with empirical results indicating that AMPpred-EL surpassed other state-of-the-art methods. In this study, the value of num_leaves was set to 31, with a learning rate of 0.05, and max_bin was set to 255.

#### CatBoost

CatBoost is a high-performance gradient-boosting algorithm designed for machine learning tasks. This method essentially involves constructing an ensemble predictor through gradient descent within a functional space [43]. One study used the feature selection recommended by the CatBoost method, combining the feature extraction methods of Extended-Connectivity Fingerprint (ECFP) with the deep neural network method to predict DPP-4 inhibitors [43]. In our study, the depth parameter was set to 6, the learning rate was set to 0.05, and the model was trained for 1,000 iterations to ensure convergence.

#### RF

RF is an ensemble machine-learning algorithm that combines the predictions of multiple decision trees to enhance accuracy and reduce overfitting [44]. Imai et al. [45] applied an RF model to a peptide database generated using BIOPEP-UWM to predict bile acid-binding peptides. Using this strategy, seven novel bile acid-binding peptides were identified. For our study, we used 150 trees and set max_features to “sqrt” to optimize the diversity and feature consideration of the models during splits.

#### Model evaluation

After the model training was completed, they were assessed using standard quantitative metrics, including accuracy (Acc), recall (Rec), precision (Pre), and F1 score (F1), defined by Eqs. 1 to 4. The receiver operating characteristic (ROC) curve visualizes the trade-off between the true-positive and false-positive rates of the model across different thresholds. The area under the ROC curve is a single scalar value that quantifies the overall performance of a model represented by the ROC curve, with higher values indicating better performance.

$$Acc=\frac{TP+TN}{TP+FP+TN+FN}$$ (1)

$$Rec=\frac{TP}{TP+FN}$$ (2)

$$Pre=\frac{TP}{TP+FP}$$ (3)

$$F1=2\times \frac{Pre\times Rec}{Pre+Rec}$$ (4)

where TN is the number of true negatives, TP is the number of true positives, FN is the number of false negatives, and FP is the number of false positives.

#### Experimental setup and runtime performance

A fivefold cross-validation was used to ensure robustness and generalization, dividing the dataset into five parts: four for training and one for testing. This process was repeated five times, each with a different test set selected to ensure that all data were tested once. External datasets were used for independent validation. The models were trained using batch gradient descent with early stopping to prevent overfitting. Crucial parameter selection was conducted using a grid-search optimization technique to identify optimal settings. The experimental environment was an Ubuntu 20.04 LTS, Intel Core i9-9900K CPU, and an NVIDIA GeForce RTX 3080 GPU. The programming was conducted using TensorFlow and PyTorch, along with key libraries such as NumPy, Pandas, and Scikit-learn for data manipulation and efficient computations in Python 3.8.

### Preparation of the prediction set

Virtual proteolysis of the proteins was conducted using the EHP-Tool program of the DFBP (http://www.cqudfbp.//enzymes/hydrolysi.com tools/dataInput.jsp). The selected proteins were the primary components of milk proteins, including casein (approximately 80% of the total protein content) and whey protein (18% of the total protein content). Casein includes α-S1-casein, α-S2-casein, β-casein, and κ-casein, while whey protein includes β-lactoglobulin and α-lactalbumin (Table S4). Three types of enzymes available in the program were selected for factors such as diversity and availability: animal proteases (trypsin, pepsin, and chymotrypsin), plant proteases (papain and stem bromelain), and microbial proteases (subtilisin). Many peptides are released via virtual proteolysis. Peptides 3 to 15 amino acids long were the primary focus. Redundancy was removed, and 737 peptides were obtained for the prediction set.

### In silico prediction

The peptides from the prediction set were inputted into the machine learning model for prediction, and the peptides with a predicted probability greater than 50% were used for the subsequent bioactivity, HIA, and toxicity predictions. The biological activities of the identified peptides were evaluated using PeptidRanker (http://bioware.ucd.ie/compass/biowareweb/Serverpages/Peptideranker.php). Peptides with a biological activity score over 0.5 were selected [46]. AdmetSAR (http://lmmd.ecust.edu.cn/admetsar1/predict/) was used to predict the HIA characteristics of the peptides. The oral absorption of small-molecule peptides occurs primarily through gastrointestinal absorption and digestion. Predicting the HIA index of active peptides helps to determine their probability of passing through the small intestine. Peptides with positive HIA values were selected for the analysis. Toxicity prediction of the identified peptides was performed using ToxinPred (http://www.imtech.res.in/raghava/toxinpred/index.html) [47]. An SVM-based prediction method with a threshold of 0.0 was chosen to predict the toxicity of peptides. Finally, 37 peptides were selected. Three peptides were randomly selected for synthesis (GPVRGPF, HPHPHL, and FVAPFPEV) by Nanjing Yuantai Biotechnology Co. Ltd. (Nanjing, China) using fluorenylmethyloxy carbonyl chloride-protected amino acid synthesis. The purity of the synthesized peptides was assessed using high-performance liquid chromatography and found to be greater than 95%.

### Determination of DPP-4 inhibitory activity

The DPP-4 inhibitory activity of the synthesized peptides was determined as previously described [48], with slight modifications. Briefly, 25 μl of the sample and 25 μl of Gly-Pro-p-nitroanilide (3.2 mM) were preincubated at 37 °C for 10 min before 50 μl of DPP-4 (0.02 U/ml) was added to the mixture (Sigma-Aldrich Co., St. Louis, MO, USA). After 60 min, the reaction was halted by adding 100 μl of 1 M sodium acetate solution. The absorbance at 405 nm was measured using a microplate reader (Thermo Fisher Scientific, Waltham, MA, USA). DPP-4 inhibition (DI) was calculated as follows:

$$DI\left(\%\right)=\left[1-\frac{A\left(\text{sample}\right)-A\left(\text{sample blank}\right)}{A\left(\text{positive}\right)-A\left(\text{negative}\right)}\right]\times 100\%$$ (5)

where *A*(sample) represents the optical density of DPP-4 in the presence of both the substrate and peptide. *A*(sample blank) represents the optical density of the 100 mM tris buffer (pH 7.6). *A*(positive) represents the optical density of the enzyme and substrate in the absence of an inhibitor. *A*(negative) represents the optical density of the enzyme in the absence of substrate and inhibitor.

### Molecular docking

Human DPP-4 (PDB ID: 5J3J) was extracted from the PDB (https://www.rcsb.org/) and processed using AutoDock Tools 1.5.6, which involved hydrogenation, charge preservation, docking atom type assignment, and pdbqt file generation for docking. The peptides were converted from their sequences to a simplified molecular input line entry system (SMILES) format using the BIOPEP-UWM database (http://www.uwm.edu.pl/biochemia/index.php/en/biopep). The SMILES format was transformed into three-dimensional molecular structures using OpenBabel software and then optimized using the MMFF94 force field. The ligand structures were processed using AutoDock Tools 1.5.6 as described above. The docking process was performed using AutoDock4 with grid dimensions set to 88 × 88 × 108 Å in the *XYZ* direction, and 50 docking runs were conducted. The interactions between peptides and proteins were analyzed using the online tool protein–ligand interaction profiler (https://plip-tool.biotec.tu-dresden.de/plip-web/plip/index) [49] and visualized using Pymol 2.5.0 and LigPlot+ 2.2.8 [50].

### Molecular dynamics simulation

Based on the molecular docking results, the peptide structures were hydrogenated to generate PDB files. All simulations were performed using Gromacs 2023.2 [51]. Amber99SB-ILDN [52] force field parameters were used to parameterize the structures of peptides and proteins and describe their interactions. The simulation system was placed in a cubic box with a side length of 108 Å and solvated with water molecules using the TIP3P water model. Sodium ions (Na^+^) were added to neutralize the charge of the system. Energy minimization was performed using the conjugate gradient (CG) method with a force cutoff of 100 kJ/(mol nm). Pre-equilibration simulations included a 0.5-ns NVT (constant number of particles, volume, and temperature) simulation and a 2-ns NPT (constant number of particles, pressure, and temperature) simulation. The simulation parameters included a temperature of 310.15 K, which was controlled using a V-rescale thermostat, and a pressure control at 1 bar using a Berendsen barostat. Long-range van der Waals interactions were truncated at 10 Å, and the particle-mesh Ewald method was used to handle electrostatic interactions. A production simulation was conducted for 100 ns with a time step of 2 fs, and snapshots were saved every 1 ps. Trajectory snapshots were obtained using Pymol 2.5.0, and structural properties such as RMSD, RMSF, Rg, and SASA were calculated using built-in tools in Gromacs.

### Statistical analysis

The experimental data were compiled using the SPSS software (version 20.0, Chicago, IL, USA). The significance of the differences between means was determined by one-way analysis of variance (ANOVA) followed by Duncan's multiple range test. Data are presented as mean ± SD. Images were created using GraphPad Prism 9 and Adobe Illustrator 2021 software.

## Data Availability

The datasets used in this paper are provided in Table S3. The source code of this work is available at https://github.com/wangliyang123/DPP-IV.git.
