# Supplementary material for: Mining Bovine Milk Proteins for DPP-4 Inhibitory Peptides Using Machine Learning and Virtual Proteolysis
Source: Research (Wash D C). 2024 Jun 17;7:0391. doi: 10.34133/research.0391 (PMC11182572; doi:10.34133/research.0391)
Supplement: Supplementary 1 — Tables S1 to S4 [file research.0391.f1.docx]

Supplementary Materials

Supplemental Table 1. Evaluations indexes of machine learning models

| Models | Acc | Rec | Pre | F1 | AUC |
| --- | --- | --- | --- | --- | --- |
| GBDT | 85.05±1.30 | 87.67±3.16 | 83.26±1.35 | 85.36±1.50 | 0.92±0.01 |
| XGBoost | 83.94±0.79 | 89.30±1.17* | 79.91±1.26 | 84.33±0.68 | 0.91±0.01 |
| LightGBM | 85.55±1.84 | 86.85±1.66 | 84.62±2.62 | 85.70±1.72 | 0.92±0.01 |
| Catboost | 81.71±1.54* | 88.04±2.68 | 78.09±1.35* | 82.73±1.59* | 0.89±0.02 |
| RF | 83.92±0.69 | 86.67±1.79 | 82.11±1.51 | 84.30±0.65 | 0.91±0.01 |

Acc, Rec, Pre, F1, and AUC represent accuracy, recall, precision, F1 score, and the area under the ROC curve, respectively. The asterisk (*) indicates a significant difference compared to the LightGBM model at a significance level of *p* < 0.05.

Supplementary Table 2: Molecular docking scoring results

| System | Binding energy (kcal/mol) | Number of  hydrophobic  interactions | Amino acid residues involved in  hydrophobic interactions | Number of  hydrogen  bonds | Amino acid residues and bond  length (Å) involved in hydrogen bonds |
| --- | --- | --- | --- | --- | --- |
| GPVRGPF-DPP4 | -9.56 | 7 | Lys122, Arg125, His126, Glu 205, Arg356, Phe358, Ile405, Tyr585 and Tyr666 | 5 | Glu206(2.54,2.51), Ser209(2.52), Arg358(2.87) and Tyr662(2.47) |
| HPHPHL-DPP4 | -10.05 | 9 | Arg125, Phe357, Ile405, Ser458, Tyr547, Tyr662, Ser630, Val656 and Tyr666 | 7 | Glu205(2.52), Arg429(2.44,2.64), Tyr456(2.84), Gln553(3.16), Tyr585(3.30) and Tyr662(2.52) |

Supplementary Table 4: Milk protein characteristics

| Name | Category | DFBP ID | Organism | Length | Mass (Da) |
| --- | --- | --- | --- | --- | --- |
| Alpha-S1-casein | casein | DFBPPR8488 | Bos taurus (Bovine) | 214 | 24529 |
| Alpha-S2-casein | casein | DFBPPR8494 | Bos taurus (Bovine) | 222 | 26019 |
| Beta-casein | casein | DFBPPR8489 | Bos taurus (Bovine) | 224 | 25107 |
| Kappa-casein | casein | DFBPPR8492 | Bos taurus (Bovine) | 190 | 21269 |
| Beta-lactoglobulin | Whey protein | DFBPPR8499 | Bos taurus (Bovine) | 178 | 19883 |
| Alpha-lactalbumin | Whey protein | DFBPPR8490 | Bos taurus (Bovine) | 142 | 16247 |

The DFBP ID is the protein identifier in the DBP database. Information on protein length and mass was obtained from the UniProt database (<https://www.uniprot.org/>).
